# Evaluation of the Appropriate Perioperative Antibiotic Prophylaxis in Italy

**DOI:** 10.1371/journal.pone.0079532

**Published:** 2013-11-13

**Authors:** Francesco Napolitano, Maria Teresa Izzo, Gabriella Di Giuseppe, Italo F. Angelillo, and the Collaborative Working Group

**Affiliations:** Department of Experimental Medicine, Second University of Naples, Naples, Italy; San Raffaele Scientific Institute, Italy

## Abstract

**Background:**

The appropriate use of antibiotics prophylaxis in the prevention and reduction in the incidence of surgical site infection is widespread. This study evaluates the appropriateness of the prescription of antibiotics prophylaxis prior to surgery amongst hospitalized patients in the geographic area of Avellino, Caserta, and Naples (Italy) and the factors associated with a poor adherence.

**Methods:**

A sample of 382 patients admitted to 23 surgical wards and undergoing surgery in five hospitals were randomly selected.

**Results:**

Perioperative antibiotic prophylaxis was appropriate in 18.1% of cases. The multivariate logistic regression analysis showed that patients with hypoalbuminemia, with a clinical infection, with a wound clean were more likely to receive an appropriate antibiotic prophylaxis. Compared with patients with an American Society of Anesthesiologists (ASA) score ≥4, those with a score of 2 were correlated with a 64% reduction in the odds of having an appropriate prophylaxis. The appropriateness of the timing of prophylactic antibiotic administration was observed in 53.4% of the procedures. Multivariate logistic regression model showed that such appropriateness was more frequent in older patients, in those admitted in general surgery wards, in those not having been underwent an endoscopic surgery, in those with a higher length of surgery, and in patients with ASA score 1 when a score ≥4 was chosen as the reference category. The most common antibiotics used inappropriately were ceftazidime, sultamicillin, levofloxacin, and teicoplanin.

**Conclusions:**

Educational interventions are needed to improve perioperative appropriate antibiotic prophylaxis.

## Introduction

Health care-associated infections (HCAIs) are an important public health threat that concerns the safety of patients and health care professionals since they are one of the leading causes of morbidity and mortality in industrialized and developing geographical region. Hospitalized patients who acquire infections while being treated for other conditions usually have a significantly longer length of stay in the hospital, are more likely to be in need of additional medical interventions with also an increased health care expenditure.

The distribution of HCAIs according to the site of infection indicated that the Surgical Site Infections (SSIs) are the most frequent among patients undergoing surgical procedures and they are considered an important indicator of the quality of the health care. The importance of the prevention and control of SSIs has been well recognized and the effectiveness of interventions has been extensively studied and many of them have been demonstrated as being effective, including surveillance systems, pre-operative preparation for the patient, appropriate administration of antibiotics prophylaxis before the initiation of surgery, aseptic procedures in the operating theatre, careful and skilled surgical technique, and postoperative surgical site or wound care. The use of antibiotics prophylaxis in the prevention and reduction in the incidence of SSIs is widespread and evidences have demonstrated the importance of timing of administration, selection of the agent, and duration of the prophylaxis [1]. Despite this evidence, the recommendations are not routinely followed and antibiotics are used excessively and inappropriately for the prevention of SSIs. Moreover, this is important in light of the fact that the prolonged use of advanced antibiotic agents may promote the development of bacterial resistance to antibiotics, so appropriate use of these agents is a critical issue.

Various epidemiological studies have been conducted in different countries describing the appropriateness of the prophylactic antibiotics use in clinical setting [2]–[4]. However, a small number of papers has been published on this topic in Italy and is evident that limited information is available [5]–[7]. Therefore, the purposes of the present cross-sectional epidemiological study were to evaluate the appropriateness of the prescription of antibiotics prophylaxis prior to surgery amongst hospitalized patients in Italy and to determine the factors associated with a poor adherence in this population.

## Methods

The data were collected between October 2009 and January 2012 from five randomly selected non-academic acute general public hospitals with a number of beds respectively of 904, 613, 549, 185, and 162, in the geographic area of Avellino, Caserta, and Naples (Italy). A total of 382 admitted patients undergoing surgery was randomly selected from 23 surgical wards and in particular four from 1 ENT (ear, nose, throat), 8 from 4 cardiac surgery, 159 from 5 general surgery, 52 from 2 gynecology, 40 from 2 oncology surgery, 31 from 4 orthopedics, 36 from 2 oral and maxillo-facial surgery, 13 from 2 neurosurgery, and 2 from 1 urology.

As a first step, a letter was delivered to the medical director of each selected hospital explaining the purpose of the survey, the non-compulsory nature of the study, and emphasizing the opportunity to participate in the project. Complete anonymity and confidentiality of patients’ data were guaranteed. From all hospitals it was obtained the permission to conduct the survey.

The survey design included a two-stage cluster sampling procedure. The first stage included the five randomly selected hospitals and the second stage consisted in a random sample of patients admitted to the surgical wards of the hospitals and undergoing surgery, excluding emergency procedures. Each patient was approached to request participation by three interviewers previously trained and not directly involved in patient care and written informed consent to participate was obtained from the subjects after explaining the study objectives. Written informed consent was also obtained from the next of kin, caretakers, or guardians on the behalf of the minors.

To determine the number of surgical procedures needed to sufficiently power the analysis, it was expected overall appropriate use of prophylactic antibiotics to be approximately 50%, assuming a confidence interval of 95%, a tolerable level of type-1 error of 5%. The minimum size required of the sample was estimated to be at least 384.

The medical record of each patient was reviewed by the three interviewers, and summarized on a standardized case report form. The following characteristics were collected for each patient undergoing surgery: age, gender, weight, height, admission diagnosis, smoking status, wound classification, type and duration of the surgical intervention, American Society of Anesthesiologists (ASA) Score, details of antibiotic prophylaxis including type of antibiotic agents, administration route, dosage, time, and duration, ward type, and length of hospital stay at time of survey. In certain kinds of operations, patient characteristics possibly associated with an increased risk of an SSIs include coincident remote site infections or colonization, diabetes, cigarette smoking, systemic steroid use, extremes of age, poor nutritional status, and perioperative transfusion of certain blood products.

Appropriateness of surgical antibiotic prophylaxis was assessed based on the Italian national guidelines [8]. For each surgical procedure the following items have been considered: the type of antibiotic, the timing of its administration within 60 minutes before surgical incision, and the length of the prophylaxis as a single or multiple doses of antibiotic administered within 24 hours. The antibiotic prophylaxis has been judged appropriate if the antibiotic, the timing, and the length of administration were in according to the guidelines.

The survey instrument went through a pilot phase for 30 surgical procedures to evaluate validity, content, and clarity. The protocol, the questionnaire, and the consent form of the study were approved by the Ethical Committee of the Second University of Naples.

### Statistical Analysis

The outcomes of interest were the overall appropriateness of antibiotic prophylaxis and the appropriateness of the timing of prophylactic administration of antibiotics prior to surgery amongst hospitalized patients. First, the comparisons between patients with and without an overall appropriate antibiotic prophylaxis and between patients with and without an appropriate prophylaxis according to the time of the administration have been conducted by using chi-square test for all categorical variables and Student’s *t*-test for independent samples to compare all continuous variables. The criterion to be met before any independent variable was considered for entry into an initial multivariable logistic regression model was a *p*-value of 0.25 or less obtained for each outcome variable in the univariate analysis with other predictors. Second, using the variables that were significant at *p*-value 0.25, a stepwise approach was used in the variable selection in which the significance level for entering and being retained in each model has a *p*-value respectively of 0.2 and 0.4. All tests were two-tailed and a *p*-value of 0.05 or less was defined as statistically significant.

In the multivariate logistic regression models the following independent variables were included: gender (male = 0, female = 1), age (continuous, in years), diabetes (no = 0, yes = 1), immunosuppression status (no = 0, yes = 1), low serum albumin (no = 0, yes = 1), clinical infection present on admission (no = 0, yes = 1), surgical ward of hospital stay (general = 0, specialties = 1), surgical wound classification (clean = 0, clean-contaminated, contaminated or dirty-contaminated = 1), ASA score (four categories 1 = 1, 2 = 2, 3 = 3, 4 = ≥4), type of anesthesia (general = 0, local = 1), undergoing endoscopic surgery (no = 0, yes = 1), implant of prosthesis (no = 0, yes = 1), and length of surgery (continuous, in minutes). The results of the multivariable models are expressed as odds ratio (OR) with 95% confidence interval (CI_95%_) and *p*-values. Statistical analysis was performed by using Stata Statistical Software (Version 10.1) [9].

## Results

All 382 selected patients agreed to participate for a response rate of 100%. A total of 404 surgical procedures were performed, with 18 and 2 patients who underwent respectively two and three procedures. The main characteristics of the sample are listed in Table 1. More than half was female, the average age was 54.2 years (14–94), half of the sample had been admitted in general surgical wards, 16.6% were diabetics, 15.1% showed low serum albumin, only 7.7% had a clinical infection on admission, and 16.1% had a ASA score 4/5. More than half of the operation based on wound surgery was classified as clean-contaminated (class II) and approximately two thirds of the patients had undergone surgery under general anesthesia. Among the patients who have received appropriate antibiotic prophylaxis, they were mainly in surgical specialties, had a surgical wound classified as clean, had an ASA score ≥3, and had undergone general anesthesia. Whereas among those who have received appropriate timing of antibiotic administration, the majority were in general surgery wards, had a surgical wound classified as clean-contaminated, had an ASA score of 2, and had undergone general anesthesia.

**Table 1:** Characteristics of the patients and of the surgical procedures associated with appropriate surgical antibiotic prophylaxis.

	Surgical procedures		Appropriate antibiotic prophylaxis		Appropriate timing of antibiotic administration	
	*n = *404		*n* = 73		*n* = 165	
*Gender*	*n*	*%*	*n*	*%*	*n*	*%*
Male	179	44.3	35	19.5	77	43
Female	225	55.7	38	16.9	88	39.1
			χ^2^ = 0.48; 1 df; *p* = 0.49		χ^2^ = 3.06; 1 df; *p* = 0.08	
*Age, years*	54.2±19.5 (14–94)ˆ		60.6±20.3 (14–94)ˆ		57.1±20.3 (14–94)ˆ	
			t = −2.83; 402 df; *p* = 0.005		t = −2.37; 307 df; *p* = 0.02	
*Diabetes*						
Yes	67	16.6	13	19.4	22	32.8
No	337	83.4	60	17.8	143	42.4
			χ^2^ = 0.09; 1 df; *p* = 0.76		χ^2^ = 1.69; 1 df; *p* = 0.19	
*Immunosuppression status*						
Yes	18	4.5	8	44.4	9	50
No	381	95.5	65	17.1	156	40.9
			χ^2^ = 8.85; 1 df; *p* = 0.003		χ^2^ = 2.34; 1 df; *p* = 0.13	
*Low serum albumin*						
Yes	61	15.1	26	42.6	22	36.1
No	346	84.9	47	13.6	143	41.3
			χ^2^ = 29.3; 1 df; *p*<0.0001		χ^2^ = 0.97; 1 df; *p* = 0.32	
*Clinical infection*						
Yes	31	7.7	14	45.2	10	32.2
No	373	92.3	59	15.8	155	41.5
			χ^2^ = 16.65; 1 df; *p*<0.0001		χ^2^ = 1.91; 1 df; *p* = 0.17	
*Ward of hospital stay*						
General Surgery	202	50	31	15.3	87	43.1
Surgical Specialties	202	50	42	20.8	78	38.6
			χ^2^ = 2.02; 1 df; *p* = 0.15		χ^2^ = 5.33; 1 df; *p* = 0.02	
*Surgical wound classification*						
I	145	35.9	42	29	65	44.8
II	228	56.4	25	11	92	40.3
III/IV	31	7.7	6	19.3	8	35.8
			χ^2^ = 19.4; 2 df; *p*<0.0001		χ^2^ = 2.12; 2 df; *p* = 0.35	
*ASA score*						
I	73	18	15	20.5	29	39.7
II	138	34.2	12	8.7	58	42
III	128	31.7	23	18	55	42.9
IV/V	65	16.1	23	35.4	23	35.4
			χ^2^ = 21.6; 3 df; *p*<0.0001		χ^2^ = 0.19; 3 df; *p* = 0.98	
*Type of anesthesia*						
General	283	70	43	15.2	114	40.3
Local	121	30	30	24.8	50	41.3
			χ^2^ = 54.66; 1 df; *p* = 0.03		χ^2^ = 0.09; 1 df; *p* = 0.76	
*Endoscopic surgery*						
Yes	107	26.5	12	11.2	25	23.3
No	297	73.5	61	20.5	140	47.1
			χ^2^ = 4.62; 1 df; *p* = 0.03		χ^2^ = 23.5; 1 df; *p*<0.0001	
*Implant of prosthesis*						
Yes	91	22.5	28	30.8	41	45
No	313	77.5	45	14.4	124	39.6
			χ^2^ = 12.8; 1 df; *p*<0.0001		χ^2^ = 0.29; 1 df; *p* = 0.59	
*Length of surgery, minutes*	119.5±94.1 (10–710)ˆ		119.5±94.1 (10–710)ˆ		133.2±112.4 (20–710)ˆ	
			t = −0.76; 367 df; *p* = 0.44		t = −1.98; 285 df; *p = *0.048	

**ˆ** Mean ± standard deviation (range).

Antibiotic prophylaxis was provided in the 81.4% of the procedures and the most commonly antibiotics used were ceftazidime (23%), levofloxacin (17.6%), and sulbactam (17.6%). The type of antibiotic administered was appropriate only in 103 surgical procedures (25.5%), and the antibiotics most frequently used inappropriately were ceftazidime, sultamicillin, levofloxacin, and teicoplanin.

Antibiotic prophylaxis for surgical procedures was appropriate only in 18.1% of cases. The results of the statistical bivariate analysis showed that patients who had received an appropriate prophylaxis were older (t = −2.83; 402 df; *p* = 0.005), with an immunosuppression status (χ^2^ = 8.85; 1 df; *p* = 0.003), with a low serum albumin (χ^2^ = 29.3; 1 df; *p*<0.0001), and with a clinical infection (χ^2^ = 16.65; 1 df; *p*<0.0001). Regard to other risk factors related to surgery, the appropriate prophylaxis was also associated with a local anesthesia (χ^2^ = 4.66; 1 df; *p* = 0.03), with a wound classified as clean (χ^2^ = 19.4; 2 df; *p*<0.0001), with the endoscopic surgery (χ^2^ = 4.62; 1 df; *p* = 0.03), and with an implant of prosthesis (χ^2^ = 12.8; 1 df; *p*<0.0001). Multivariate logistic regression analysis was performed to assess the impact of a number of factors on the likelihood that patients had received an appropriate antibiotic prophylaxis. Of the variables considered in the analysis, level of serum albumin, clinical infection, ASA score, and classification of the operation based on wound surgery were found to be significantly and independently associated with the appropriate antibiotic prophylaxis (Model 1 in Table 2). The strongest predictors of appropriate antibiotic prophylaxis were related to the patients’ health status, since those having a low serum albumin (OR = 3.66; CI_95%_ 1.8–7.44) and a clinical infection (OR = 3.9; CI_95%_ 1.56–9.75) were almost four times as likely as those who did not have a low serum albumin and a clinical infection to receive an inappropriate antibiotic prophylaxis. Moreover, appropriate antibiotic prophylaxis was more frequently observed in patients with a wound classified as clean (OR = 0.32; CI_95%_ 0.17–0.61). Compared with patients who had an ASA score ≥4, those with an ASA score 2 were correlated with a 64% (OR = 0.36; CI_95%_ 0.17–0.77) reduction in the odds of having an appropriate antibiotic prophylaxis.

**Table 2:** Profiles for appropriate antibiotic prophylaxis prior to surgery amongst hospitalized patients using multivariate logistic regression analysis.

Variable	OR	SE	CI_95%_	*p* value
Model 1. Appropriate antibiotic prophylaxis				
Log likelihood = −153.07, χ^2^ = 70.98 (8 df), *p*<0.00001				
Low serum albumin	3.66	1.32	1.8–7.44	<0.0001
Wound type clean	0.32	0.11	0.17–0.61	0.001
Clinical infection	3.9	1.82	1.56–9.75	0.004
ASA score				
≥IV	1.0*	–	–	–
II	0.36	0.14	0.17–0.77	0.008
III	0.66	0.22	0.34–1.28	0.22
Immunosuppression status	2.73	1.58	0.88–8.46	0.08
Implant of prosthesis	1.74	0.59	0.9–3.37	0.1
Endoscopic surgery	0.59	0.23	0.28–1.27	0.18
Model 2. Appropriateness of the timing of prophylactic administration of antibiotics				
Log likelihood = −166.72, χ^2^ = 56.19 (9 df), *p*<0.00001				
Endoscopic surgery	0.16	0.06	0.08–0.31	<0.0001
General surgery wards	0.34	0.11	0.19–0.63	0.001
Length of surgery	1.01	0.001	1.0003–1.01	0.028
Age	1.02	0.01	1.001–1.03	0.037
ASA class				
≥IV	1.0*	–	–	–
I	2.39	1.05	1.02–5.64	0.05
II	1.4	0.49	0.71–2.8	0.33
Immunosuppression status	4.44	3.78	0.84–23.6	0.08
Diabetes	0.54	0.22	0.25–1.18	0.12
Clinical infection	1.98	1.31	0.54–7.26	0.3

* Reference category.

The evaluation of the appropriateness of the timing of prophylactic administration of antibiotics, defined as only an injection occurred within 60 minutes before surgical incision, indicates an appropriateness only in 53.4% of the surgical procedures. The bivariate associations between variables of interest and appropriate timing of prophylactic administration of antibiotics are reported in Table 1. The appropriate timing of antibiotic administration was associated with patients older (t = −2.37; 307 df; *p* = 0.02), with admission to general surgery wards (χ^2^ = 5.33; 1 df; *p* = 0.02), with not undergoing endoscopic surgery (χ^2^ = 23.5; 1 df; *p*<0.0001), and with a higher length of surgery (t = −1.98; 285 df; *p* = 0.048). The results of the multivariate logistic regression model with the ORs for the outcome of interest by the different variables showed that those that were identified in the univariate analysis retained their statistical significance (Model 2 in Table 2). Indeed, an appropriate time of the administration rate was more frequently observed in older patients (OR = 1.02; CI_95%_ 1.01–1.03), in those admitted in general surgery wards (OR = 0.34; CI_95%_ 0.19–0.63), in those who did not undergo endoscopic surgery (OR = 0.16; CI_95%_ 0.08–0.31), and in those with a higher length of surgery (OR = 1.01; CI_95%_ 1.0003–1.01). Moreover, an appropriate prophylaxis was observed more likely in patients with an ASA score 1 (OR = 2.39; CI_95%_ 1.02–5.64) when ASA score ≥4 was chosen as the reference category.

## Discussion

This study is a comprehensive assessment of the prevalence of appropriate prescription of antibiotics prophylaxis prior to surgery amongst hospitalized patients and of the factors that may influence compliance in a sample that has not been previously surveyed in Italy. In this current study it has been found a substantial proportion of inappropriate surgical antibiotic prophylaxis in accordance with recommendations. The surgical antibiotic prophylaxis was administered in line with local recommendations in less than 20% of patients in this cohort. This finding is in accordance with other studies conducted in different countries. In a large study conducted in France, with 19.4% of the procedures received surgical antibiotic prophylaxis in complete compliance regarding antibiotic choice, timing of first dose, and total duration of time [3]. The results observed differ substantially from existing scientific literature. Indeed, two cross-sectional studies carried out in tertiary referral teaching hospital in Italy and in a tertiary care private hospital in India found that antibiotic appropriate prophylaxis was provided respectively in 44.8% [10] and in 52% [11] of patients. A study conducted in seven hospitals in Germany observed that the guidelines for antibiotic prophylaxis were followed in 70.7% of the patients underwent surgery [12] and another survey in sixty two acute-care hospitals in Japan showed that the inappropriateness rates for drug selection and treatment duration during general surgical procedures were 16–47% and 32–62%, respectively, depending on surgical procedures [13]. A study held in a General Surgery Clinic in a hospital in Greece showed that 78.5% of the surgical procedures required prophylaxis, but it was administered in 97.5%, so it was inappropriately administered in 19% [2]. Two other investigations in Canada and in Australia found that almost all (93%) patients in the setting of surgical treatment received an appropriate preoperative dose of antibiotic for closed fractures [14] and for prosthetic knee and hip joint replacements [15], respectively. By contrast, compliance with antibiotic prophylaxis in a Brazilian hospital in adult patients undergoing orthopedic, neurologic, and cardiac surgeries showed a compliance index of 5.8%, 3.1%, and 3%, respectively [16]. Regarding the appropriateness of the timing of prophylactic administration of antibiotics, the value observed in the present study (53.4%) was considerably lower than those found in already mentioned surveys with the timing of prophylaxis administration that was appropriate respectively in 100% [2], 89% [11], 86.4% [17], 84% [6], 83% [4], 76.6% [3], and 75.7% [10] of the monitored patients.

In the present study the most commons antibiotics used were ceftazidime, levofloxacin, sultamicillin, and teicoplanin. These results differ substantially from those observes in similar previous studies [2], [4], [11], [12]. Previous experiences in Italy showed that the most common antibiotics used for prophylaxis in a tertiary teaching hospital were β-lactam/β-lactamase inhibitors, cefazolin, and third-generation cephalosporin [10], and in a large number of hospitals cefazolin, amoxicillin-clavulanic acid, and sultamicillin [6].

Comparison of the present results with those from previously published research should be done with caution, because it is important to emphasize that visible discrepancies in some of the observed findings are probably partly attributable to different composition of the study populations, differences in the methodologies used, and in the criteria used to assess the adherence to recommendations for appropriate antibiotic prophylaxis. There is no doubt that the present survey suggest that hospital managers and clinicians in hospitals should be involved in promoting efficient and appropriate initiatives and the application of clinical guidelines are of paramount and immediate importance and will be useful in decreasing inappropriate surgical antibiotic prophylaxis. Moreover, it was of particular concern the finding observed since it is well-established that if the administration of the antibiotic prophylaxis does not initiate at the appropriate time the patient had an increased risk of surgical site infection [18]–[20].

In this study it was possible to identify, according to the multivariate models, that several important factors emerged as being significantly associated with the appropriate use of antibiotic prophylaxis. In particular, several patients’ characteristics, such as older age, having hypoalbuminemia, having a clinical infection, having a type of wound clean, contaminated or dirty-contaminated, having been admitted in general surgical wards, not having been underwent an endoscopic surgery, having a higher length of surgery, and having an ASA score 1, were highly predictive of having an appropriate antibiotic prophylaxis. A previous study conducted in Italy showed that in multivariate logistic regression model an inappropriate prophylaxis was more frequently in patients with ASA score ≥2 and with a longer length of surgery, whereas those undergoing endoscopic surgery and with a surgical wound classification ≥2 received less frequently an inappropriate prophylaxis [7]. In the already mentioned study performed in France, antibiotic choice and duration of prophylaxis were significantly associated, although in the univariate analysis, with younger age, a higher surgical risk infection, pre-operative hospital stay >48 hours, and multiple procedures performed [3]. In Japan, inappropriate antibiotic selection and treatment duration were more likely in patients undergoing a higher number of surgical procedures and a laparoscopic cholecystectomy [13].

When interpreting the findings of this study, there are some potential limitations that should be noted. First, cross sectional surveys are limited by capturing only a moment in time, and the understanding regarding the directionality of the reported associations is limited by reverse causality, a feature of this type of study design. Second, the design of the study implicates that adherence to prescribing guideline-discordant prophylaxis was only taken into account when it was recorded in the patients’ medical charts. Third, the study results may reflect the epidemiology and guideline adherence across hospitals. However, the goals of the study were to provide an overview of the antibiotic prophylaxis guideline adherence and the appropriateness of prescribed prophylaxis among all patients undergoing surgical treatment, and we do believe that the data provide insights into daily clinical practice. The reasons for non-adherence to antibiotic prophylaxis guidelines were beyond the scope of the current study. Despite the limitations, these data are highly important because they provide information that contribute to the understanding of the appropriateness of the prescription of antibiotics prophylaxis prior to surgery and the factors associated amongst hospitalized patients in Italy and the extremely high response rate provided a robust data set for the general purposes of this study.

In conclusion, the results provide evidence that health care providers should be aware of their larger role in reducing unnecessary and inappropriate prescription of antibiotics prophylaxis in patients prior to surgery. An optimum utilization of medical assistance resources represent an important challenge for health service providers in order to improve the efficiency of healthcare systems and to contribute to the prevention and control of SSIs and there is a clear need for additional efforts and educative interventions to improve antibiotic prophylaxis.
